# Embedding Culture of Care to Improve Safety and Patient Experience and to Reduce Restrictions on a Forensic Medium Secure ICU

**DOI:** 10.1192/bjo.2026.11480

**Published:** 2026-06-30

**Authors:** Syed Mohsin Ali Shah, Leela Sivaprasad, Onaiza Awais, Michael Nti-wireko, Priya Gilbert

**Affiliations:** Birmingham And Solihull Mental Health NHS Foundation Trust, Birmingham, United Kingdom

## Abstract

**Aims:**

This QI project aimed to implement the NHS England Culture of Care framework on Severn Ward, a forensic PICU and National pilot site.

Objectives were to:

(1) To reduce restrictive practices

(2) Enhance patient and staff experience through Culture of Care principles and lived experience.

(3) Improve the therapeutic environment and relational safety.

**Methods:**

Between January 2024 and December 2025, a structured multidisciplinary QI approach was implemented using co-production and PDSA cycles. Patient voice was central to the project through ward representatives, discussions and recovery suppers, and co-led morning meetings to shape daily activity choices and ward expectations.

Key interventions included:

• De-escalation space/sensory room being set up.

• New Gold, Silver, Bronze sharps system to support proportionate safety measures.

• Expanded therapeutic activity programme (gardening, art therapy, drum circle, breakfast club).

• Allowing community leaves pathways from PICU (removing blanket restriction on leaves).

• Access to mobile phones.

• Environmental improvements: new examination room, new ward gym, moving CTM to a more welcoming room with windows, increased courtyard access, opening up dining room.

• Staff induction leaflet promoting wellbeing and relational safety.

• Embedding Autism Informed Care principles (sensory regulation, communication needs, SALT involvement).

Due to the patient cohort, qualitative feedback from patients and staff was prioritised over quantitative data. Patient and staff interviews and questionnaires were completed, patients on Rehabilitation wards with prior experience of the ICU acted as Expert by experience with regards the changes they would like to see in the PICU setting.

**Results:**

Service users consistently reported feeling more heard, respected, and involved, especially through co-leading morning meetings. This improved transparency and shared ownership of ward life. Patients described increased privacy and freedom of movement, reduced boredom and more meaningful activity engagement. The new ward gym also helped in patients having better access to physical exercise to regulate their emotions and taking care of their physical health.

Staff reported a calmer atmosphere which is less reliant on blanket restrictions. They also felt more confident in applying Trauma-Informed and Autism-Informed approaches. The de-escalation room became a valued early intervention tool. Environmental improvements enhanced dignity and therapeutic interaction. Staff also noted improved team cohesion and clearer shared values.

Organisational barriers slowed completion of some interventions.

**Conclusion:**

Qualitative evidence from staff and patients strongly indicates positive cultural change despite variability in patient acuity. Sustained progress will require continuedorganisational support and ongoing PDSA cycles to keep Culture of Care at the centre of patient care.

